# A Rare Presentation of Jejunojejunal Intussusception in an Adult: A Case Report

**DOI:** 10.7759/cureus.89719

**Published:** 2025-08-10

**Authors:** Moh'd Obeidat, Hajem Abu dalu, Mohammad Atrooz, Bilal Rafiq, Tawheed Alsmadi, Mohammad Btainah, Tha'ir Al-Tarabsheh, Wisam Al-Sukkar

**Affiliations:** 1 General Surgery, The Royal Medical Services (King Hussein Medical Center), Amman, JOR; 2 ٌRadiology, The Royal Medical Services (King Hussein Medical Center), Amman, JOR; 3 Anaesthesiology, The Royal Medical Services (King Hussein Medical Center), Amman, JOR; 4 Anaesthesiology, King Hussein Medical City, Amman, JOR; 5 Obstetrics and Gynaecology, King Hussein Medical City, Amman, JOR; 6 Surgery, The Royal Medical Services (King Hussein Medical Center), Amman, JOR; 7 Anaesthesiology, King Hussein Medical Center, Amman, JOR

**Keywords:** adult, intussusception, jejunum, laparotomy, vomiting

## Abstract

Jejunojejunal intussusception is a rare surgical condition. It is an abnormal invagination of a part of the jejunum into the section immediately ahead of it. It can be caused by enlarged lymph mode, polyps, viral infections and malignancies. We report a case of jejunojejunal intussusception caused by hamartomatous polyp in 24-year-old female patient who presented with chronic abdominal pain and vomiting. The patient's medical history, physical examination and radiological testing helped us reach the diagnosis of jejunojejunal intussusception. The patient underwent laparotomy, manual reduction of the affected small bowel and resection of the bowel containing the intraluminal mass. The recovery was uneventful.

## Introduction

Jejunojejunal intussusception in adults due to hamartomatous polyp is an extremely rare pathological disorder and only a few cases are reported in the literature [1-12]. Intussusception in general can be described as a telescoping of a segment of the gastrointestinal tract (the intussusceptum) into an adjacent distal segment (the intussuscipiens), leading to obstruction and compromised blood flow. It is broadly categorized anatomically into four types: enteroenteric (small intestine into small intestine), ileocolic (terminal ileum into colon), ileocecal and colocolic [7]. Adult intussusception is estimated to be 5% of all reported intussusceptions [2]. It can be idiopathic or caused by benign or malignant conditions. Regarding sex distribution, studies have shown no consistent sex predominance, although some reports suggest a slight male predominance in adults [7,9]. The usual clinical presentations of intussusception are abdominal pain or discomfort and vomiting. The non-specific signs and symptoms make the diagnosis of adult intussusception preoperatively very difficult or delayed. As many as 90% of all adult intussusceptions require surgical intervention [3,4]. A combination of a good clinical examination, blood tests, radiological studies and the type of surgery can be helpful for a better outcome. Here we report a case of jejunojejunal intussusception that was successfully treated with segmental resection of the involved part.

## Case presentation

A 24-year-old woman presented to our general surgery clinic in May 2023 with a history of abdominal pain and non-bilious vomiting for the past year. Her past medical history included laparoscopic cholecystectomy in 2022 for symptomatic gallstone disease, with preoperative ultrasonography confirming cholelithiasis and no evidence of small bowel pathology. She described the abdominal pain as intermittent, dull aching in nature, non-radiating and generalized across the abdomen. There were no clearly identified aggravating or relieving factors. The patient denied fever, altered bowel habits, abdominal distension, rectal bleeding, loss of appetite or trauma. She also did not experience any urinary or gynecological symptoms. All of her vital signs were normal. On clinical examination, her abdomen was soft, symmetric and non-tender without distention. Her laboratory test results were within normal limits except for hemoglobin level (10.6 g/dL), which was mildly reduced (Table 1).

**Table 1:** Laboratory test results

Test Name	Patient's serum level	Normal range	Unit
White Blood Cells	8.2	3.4-9.6	10^3^/uL
Red Blood Cells	4.99	3.92-5.13	10^6^/uL
Hemoglobin	10.6	11.6-15	g/dL
Hematocrit	33.3	35.5-44.9	%
Platelets	245	157-371	10^3^/uL
Prothrombin Time (PT)	13.6	13.4-16.1	s
International Normalized Ratio (INR)	1.02	0.8-1.2	
Alanine Aminotransferase (ALT)	24.3	≤41	U/L
Aspartate Transferase (AST)	19.1	≤37	U/L
Alkaline phosphatase (ALP)	109	40-129	U/L
Creatinine	0.76	0.5-1.2	mg/dL
Blood Urea Nitrogen (BUN)	9.4	6-20	mg/dL
Glucose Serum	103.8	70-110	mg/dL
Sodium	137	135-153	mEq/L
Potassium	4.1	3.5-5.5	mEq/L
Calcium	9.66	8.4-10.5	mg/dL
Phosphorus	3.78	2.5-4.5	mg/dL
Uric Acid	4	2.7-6.1	mg/dl
Albumin	46.1	35-55	g/L
Amylase	80	28-100	U/L
Bilirubin Total	0.543	0.1-1.2	mg/dL
Bilirubin Direct	0.209	0.01-0.3	mg/dL

**Figure 1:**
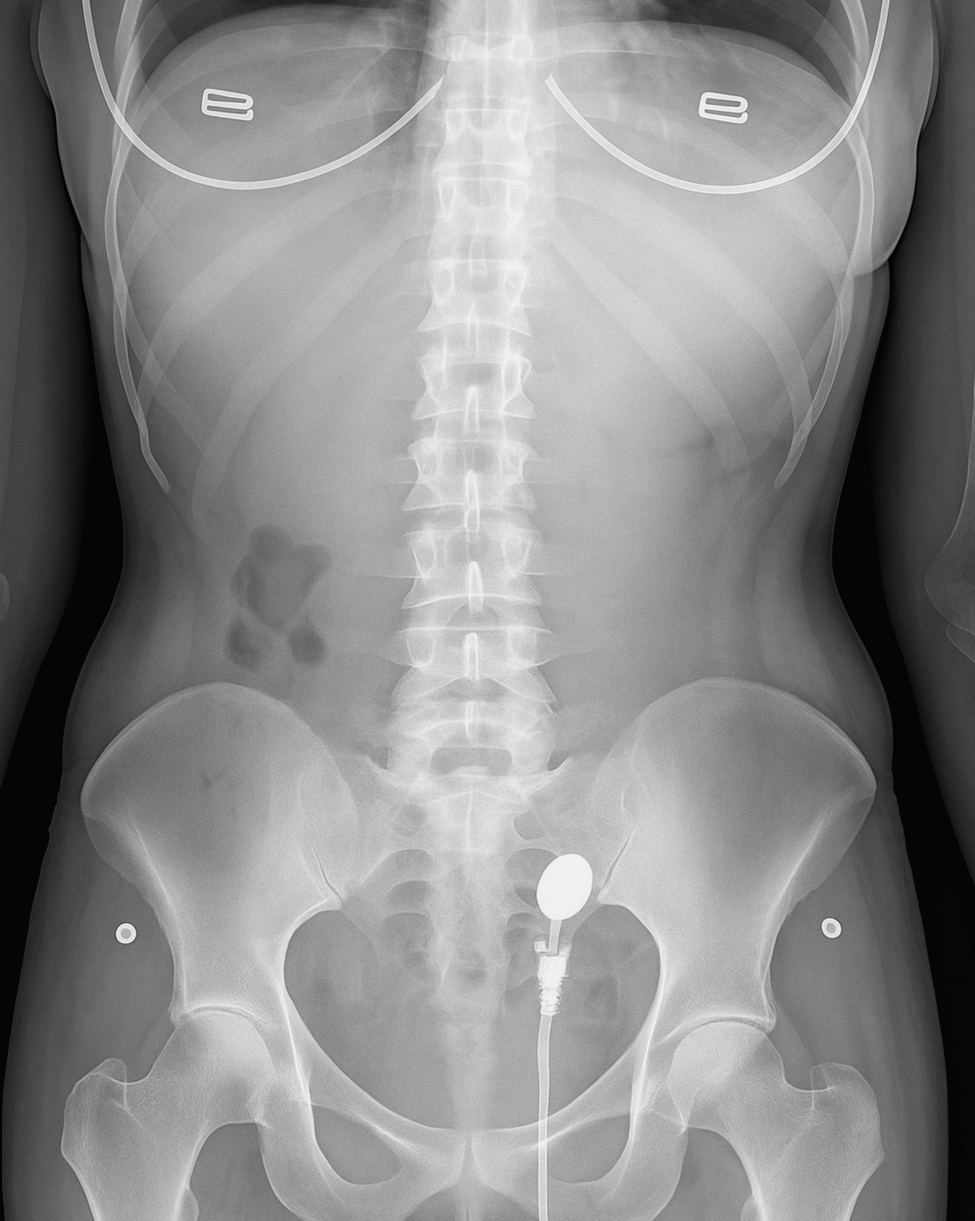
plain abdomen X-ray of the patient

The lower hemoglobin level was attributed to menstrual blood loss, as the patient reported regular cycles with moderately heavy flow. Plain abdomen x-ray was normal (Figure 1).

**Figure 2:**
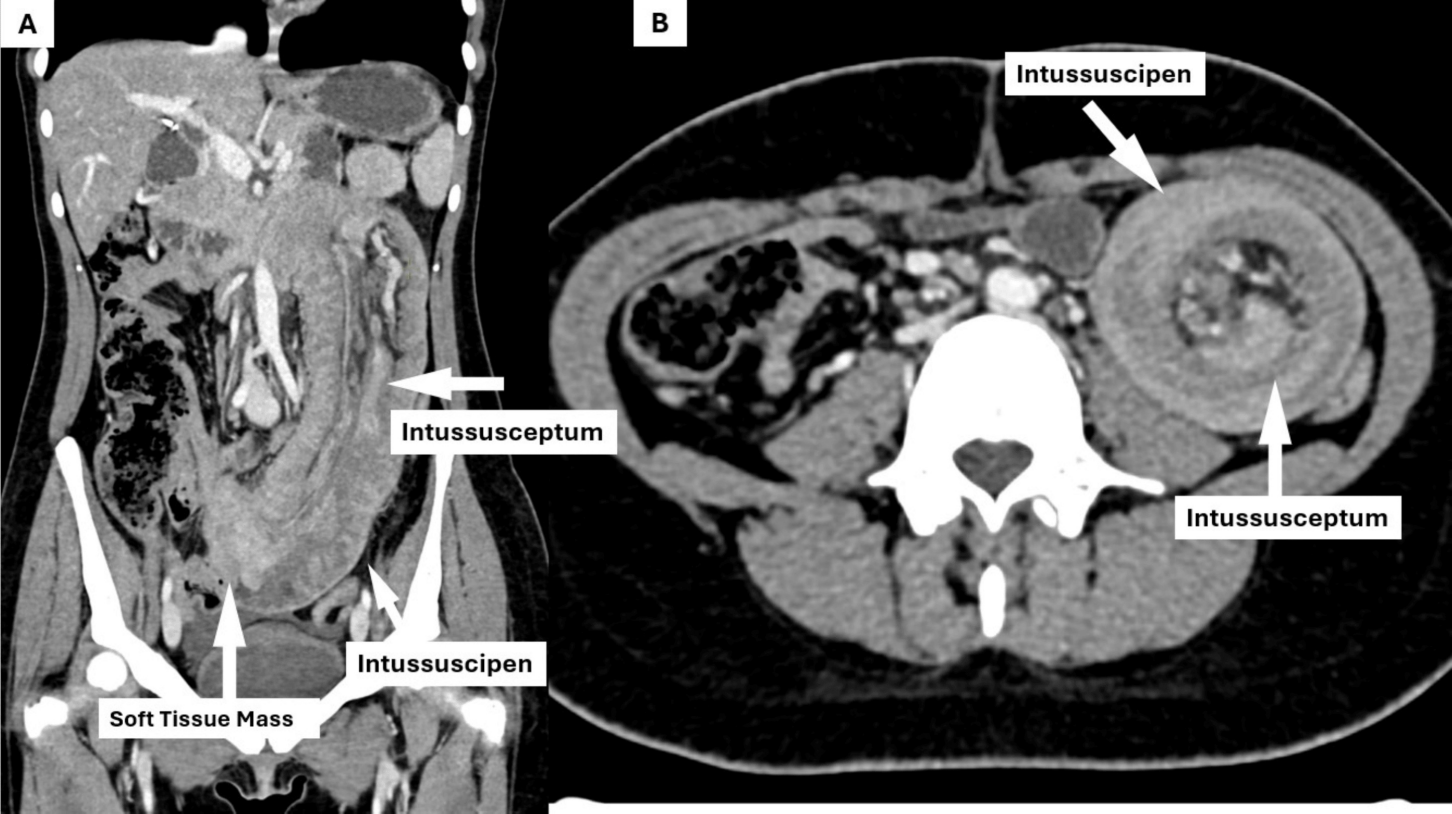
CT scout films showing the intussusceptum, intussuscipen and the soft tissue mass (leading point) (A) Coronal CT image demonstrating a long segment of small bowel intussusception. The intussuscipiens and intussusceptum are labeled, along with a soft tissue mass acting as the lead point. (B) Axial CT image demonstrating the characteristic “target sign” of intussusception, with the intussuscipiens forming the outer bowel wall and the intussusceptum visible centrally.

A computed tomography (CT) scan showed a segment of jejunal intussusception, where the outer bowel loop (intussuscipiens) enveloped the inner loop (intussusceptum) along with its associated mesenteric fat, vessels and lymph nodes. Additionally, a lobulated, enhancing soft tissue mass was identified in the mid-abdomen, serving as the lead point for the intussusception (Figure 2A, 2B).

**Figure 3:**
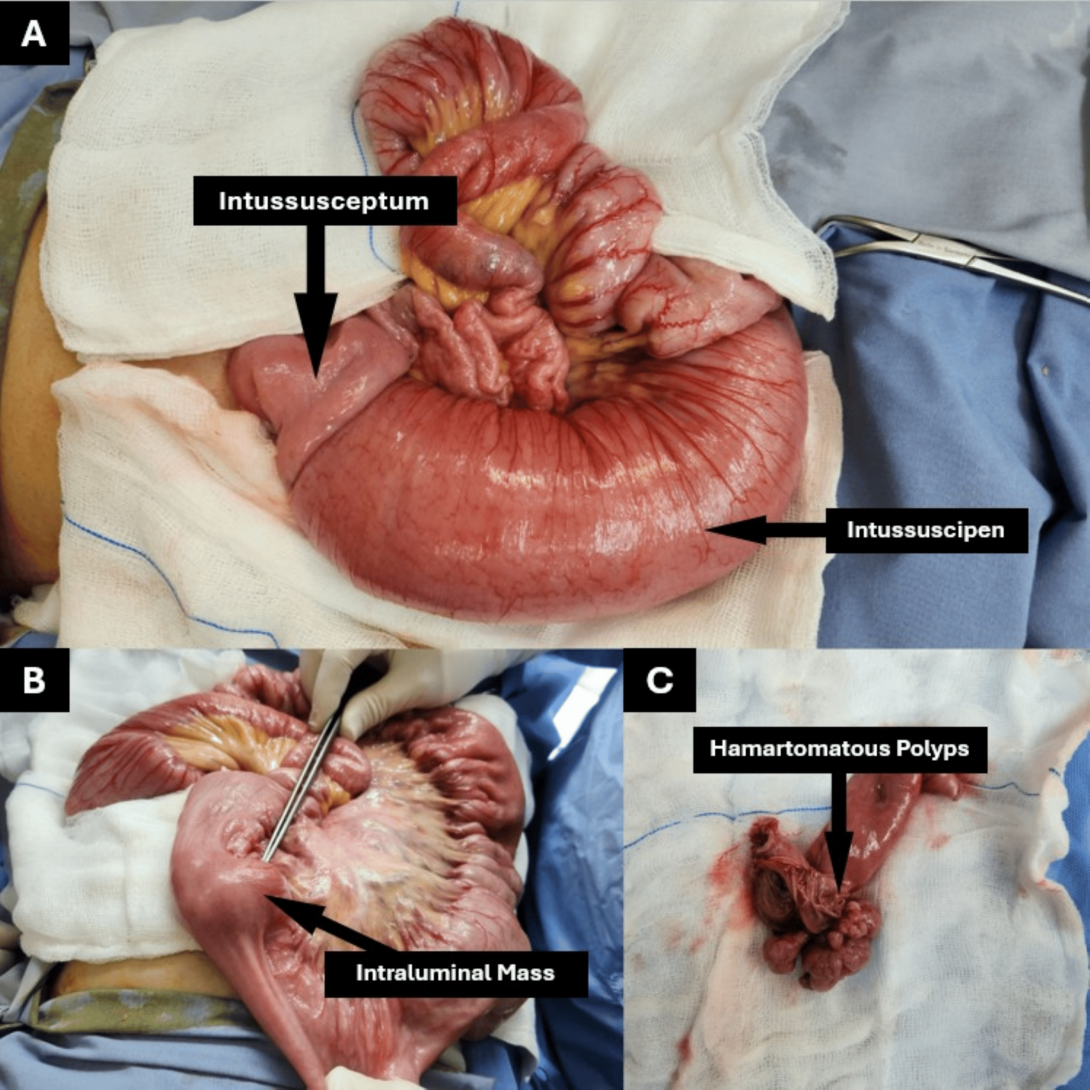
Intraoperative findings include the intussusception and intraluminal soft tissue mass. (A) Intraoperative view showing jejunojejunal intussusception with the intussuscipiens and intussusceptum clearly visible. (B) Intraoperative image after reduction revealing an intraluminal mass at the lead point. (C) Resected specimen showing multiple hamartomatous polyps on the opened bowel segment.

Given the patient's age, sex and prior surgical history, a thorough differential diagnosis was considered. Adhesive small bowel obstruction was considered due to her history of laparoscopic cholecystectomy; however, CT imaging did not show signs of adhesive obstruction. Laboratory investigations showed normal liver enzymes and pancreatic enzymes, making post-cholecystectomy complications and chronic pancreatitis unlikely. Based on clinical, laboratory and imaging findings, a diagnosis of jejunojejunal intussusception with a possible intraluminal lead point was established. The patient was scheduled for open surgery the following day after obtaining her informed consent. Laparoscopic reduction was not attempted due to the patient’s prior laparoscopic cholecystectomy, which raised concern for adhesions, and the presence of a suspected intraluminal mass on CT imaging. These factors warranted an open approach to facilitate safe resection and accurate assessment of potential malignancy. At the time of surgery, an exploratory laparotomy was performed through a midline incision. Intraoperative findings revealed jejunal intussusception located approximately 30 cm distal to the duodenojejunal (DJ) junction (Figure 3A). A small intraluminal mass was palpable just proximal to the site of intussusception (Figure 3B, 3C).

The patient underwent manual reduction of the involved segment, followed by resection of a 15-cm portion of the jejunum containing the intraluminal mass, with subsequent primary side-to-side re-anastomosis using a linear stapler. The patient was extubated immediately after surgery and sent to the female surgical ward. Oral intake was initiated on the second postoperative day, and the patient was discharged home two days thereafter. At her two-week follow-up, she was completely asymptomatic with a healed wound. Pathological analysis revealed a segment of small bowel measuring 7.5 cm with intussuscepted part measuring 5 cm, showing polypoidal fungating tumor measuring 4.5 m in axial dimension, located 2.5 cm and 7 cm from axial surgical margins. The tumor was surrounded by serosal tethering. Histopathological examination confirmed the complete excision of a hamartomatous polyp with clear resection margins and no evidence of dysplasia or malignancy. Two lymph nodes were identified, both exhibiting features of non-malignant reactive response.

## Discussion

Adult jejunojejunal intussusception is extremely rare [1-12]. Adult intussusception accounts for only 5% of all reported cases of intussusception, and jejunojejunal involvement is a rare subset. In comparison, over 90% of intussusception cases occur in the pediatric age group [5,7]. Intussusception is described as an extraordinary invagination or telescoping of a proximal segment of the intestine (intussusceptum) into an adjacent distal segment (intussuscipiens). Intussusception in general was first described in 1674 by Barbette of Amsterdam [7]. There is a major difference between adult and pediatric intussusception regarding etiology, clinical presentation and management. Almost all pediatric intussusceptions are idiopathic in nature (90%), while most adult intussusceptions have a lead point. The leading point can be benign or malignant pathology like polyps, viral infections, strictures, adhesions or malignancies [2-4,8].

There are a wide variety of clinical presentations mentioned in the literature for intussusception, but there is a huge difference between adults and pediatric age groups. In children, the symptoms are colicky abdominal pain, lethargy, fever and currant jelly-like stools [3]. On the other hand, symptoms in adults are usually nonspecific like abdominal pain/discomfort, vomiting, diarrhea, constipation, abdominal distension, loss of weight and rectal bleeding [8]. The previously mentioned nonspecific symptoms can lead to a delay or even miss the diagnosis of adult intussusception [5].

The diagnosis of adult intussusception is challenging for any surgeon. Plain abdomen x-ray and barium swallow and enema are good radiological modality to start with if obstruction is suspected with a low accuracy rate [3]. A CT scan with intravenous contrast (IV) contrast is the best diagnostic tool for detecting adult intussusception with high sensitivity. It is also good to determine the causes of intussusception [3]. Ultrasound is a less sensitive modality than the CT scan. In some cases, it can be used to visualize the target sign.

The treatment of intussusception depends on many factors like the patient’s age, the cause of intussusception and the presence of signs of bowel ischemia. Supportive measures like hydration, electrolyte correction, nutritional support and vitamin supplementation should be initiated before any intervention. Surgical intervention with careful manual reduction±resection of the involved bowel segment is still the treatment of choice, especially in the case of malignancies [8].

## Conclusions

Adult jejunojejunal intussusception is an uncommon surgical condition with nonspecific clinical features that often delay diagnosis. In this case, the patient presented with vague abdominal symptoms and contrast-enhanced CT imaging proved critical in identifying the characteristic “target sign” of intussusception and suggesting an underlying intraluminal mass. Surgical exploration, including manual reduction with or without segmental resection of the involved bowel segment, remains the gold standard for managing adult intussusception. This case highlights the importance of maintaining a high index of suspicion and the role of timely imaging and surgical intervention in achieving a favorable outcome.
